# Supplementary material for: Identification of photoperiod-induced specific miRNAs in the adrenal glands of Sunite sheep (Ovis aries)
Source: Front Vet Sci. 2022 Jul 22;9:888207. doi: 10.3389/fvets.2022.888207 (PMC9354845; doi:10.3389/fvets.2022.888207)
Supplement: Supplementary file 1 [file Data_Sheet_1.ZIP › Supplementary file/miRDeep2 file.zip!/miRDeep2 file/Know_Structure/LP42A2/oar-mir-487a.pdf]

miRBase precursor : oar-mir-487a
Total read count : 114
oar-miR-487a-5p read count : 22
oar-miR-487a-3p read count : 89
remaining reads : 3

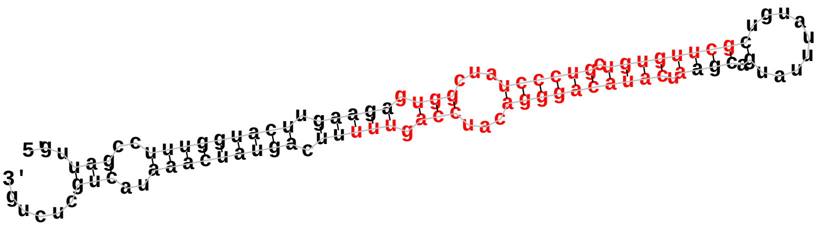

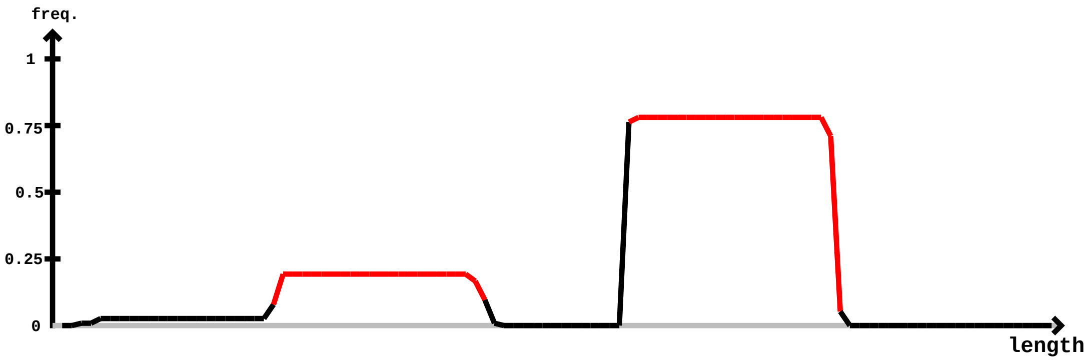


```
                                                                      oar-miR-487a-3p
                              oar-miR-487a-5p
5'- guuagccuuugguacuugaagaguggcuaucccugcuguguucgcuguauuuaugacgaaucauacagggacauccaguuuuucaguaucaaauacugcucug   -3'    exp
    ..(((..(((((((((.(((((.(((...((((((.(((((((((.........).)))).))))))))))...))).))))).)))))))))..))).....    reads     mm        sample
    .uuagccuuugguacuugaag..................................................................................    1         0         seq
    ...agccuuugguacuugaaga.................................................................................    2         0         seq
    ....................aguggcuaucccugcuguguu..............................................................    3         0         seq
    ....................aguggcuaucccugcuguguuc.............................................................    4         0         seq
    ......................guggcuaucccugcuguguuc............................................................    4         0         seq
    ......................guggcuaucccugcuguguucg...........................................................    10        0         seq
    ......................guggcuaucccugcuguguucgc..........................................................    1         0         seq
    ..........................................................aaucauacagggacauccagu........................    8         0         seq
    ..........................................................aaucauacagggacauccaguu.......................    75        0         seq
    ..........................................................aaucauacagggacauccaguuu......................    4         0         seq
    ...........................................................aucauacagggacauccaguuu......................    2         0         seq
```
